# 2D Analytical Model for the Directivity Prediction of Ultrasonic Contact Type Transducers in the Generation of Guided Waves

**DOI:** 10.3390/s18040987

**Published:** 2018-03-26

**Authors:** Kumar Anubhav Tiwari, Renaldas Raisutis, Liudas Mazeika, Vykintas Samaitis

**Affiliations:** Prof. K. Barsauskas Ultrasound Research Institute, Kaunas University of Technology, K. Baršausko St. 59, LT-51423 Kaunas, Lithuania; renaldas.raisutis@ktu.lt (R.R.); liudas.mazeika@ktu.lt (L.M.); vykintas.samaitis@ktu.lt (V.S.)

**Keywords:** analytical model, directivity pattern, guided wave, macro-fiber composite (MFC), Lamb mode, shear horizontal, ultrasonic non-destructive testing

## Abstract

In this paper, a novel 2D analytical model based on the Huygens’s principle of wave propagation is proposed in order to predict the directivity patterns of contact type ultrasonic transducers in the generation of guided waves (GWs). The developed model is able to estimate the directivity patterns at any distance, at any excitation frequency and for any configuration and shape of the transducers with prior information of phase dispersive characteristics of the guided wave modes and the behavior of transducer. This, in turn, facilitates to choose the appropriate transducer or arrays of transducers, suitable guided wave modes and excitation frequency for the nondestructive testing (NDT) and structural health monitoring (SHM) applications. The model is demonstrated for P1-type macro-fiber composite (MFC) transducer glued on a 2 mm thick aluminum (Al) alloy plate. The directivity patterns of MFC transducer in the generation of fundamental guided Lamb modes (the S0 and A0) and shear horizontal mode (the SH0) are successfully obtained at 80 kHz, 5-period excitation signal. The results are verified using 3D finite element (FE) modelling and experimental investigation. The results obtained using the proposed model shows the good agreement with those obtained using numerical simulations and experimental analysis. The calculation time using the analytical model was significantly shorter as compared to the time spent in experimental analysis and FE numerical modelling.

## 1. Introduction

The ultrasonic guided wave (UGW) technology is widely used for structural health monitoring (SHM) and non-destructive testing (NDT) of elongated structures [1,2]. Various types of guided waves (GWs) are available, such as Rayleigh or surface waves and Lamb waves which can propagate in bounded media. In comparison to other UGW testing methods, Lamb wave testing is fast and more sensitive to the surface and internal defects over the thickness of the structure. [3,4,5]. Although UGW testing is an effective technique for the inspection of defects a few meters away from the transmitting transducers, defects may be located at longer distances [6,7]. Many contact and non-contact type ultrasonic transducers are being used for the generation and reception of GWs [8,9]. The contact transducers are used to make a direct contact with the test structure. Although contact methods are limited to their scanning speed and coverage area, they are still popular due to possibilities of easy handling of a transducer, moving the transducer on the surface of the structure being inspected and covered by coupling medium and high acoustic impedance matching between the transducer and object. Moreover, the contact transducers can be easily designed, configured or adjusted depending on the type of defects or damages and the availability of access region of the structure. Thus, the contact transducers are widely used in the various field of NDT such as the detection of flaws with straight beam test, inspection of delamination or disbonds in composites and many thickness gauging applications [10,11,12,13]. In this research work, the focus was kept on the contact-type ultrasonic transducers for the generation of GWs.

The contact-type ultrasonic transducers or the transducers glued to the surface of the structure generating the GWs are required for the accurate inspection of the defects at larger propagation distances from a fixed location on the structure. For a specific medium, a transducer operating as a transmitter or a receiver has a unique directivity pattern. The transducer beam width for a particular medium is the ratio of its diameter to the operating wavelength of UGW. Thus, a transducer with larger size (diameter) produces a narrower beam in comparison to the smaller sized transducers. The major amount of wave energy is contained by the main lobe of a directivity pattern while side lobes signify the undesired radiations, reflections etc.

**Figure 1:**
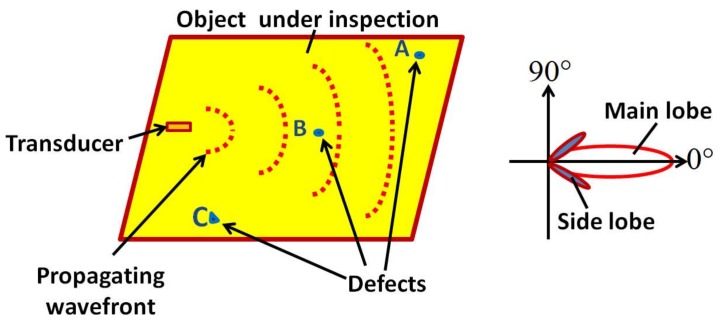
Explaining the directivity pattern of a directional transducer glued on the object under inspection.

Figure 1 shows a transducer glued on an object under inspection to demonstrate the effect of directivity pattern on the probability of the defect estimation. The directivity pattern as a function of polar angle is also illustrated to show the region of maximum radiation (main lobe) and the undesired radiations (side lobes). It can be clearly observed from Figure 1 that defect-A and defect-C cannot be inspected from the presented arrangement of the transmitting transducer as they are not in the region of coverage (directivity) of the transducer. Hence, in order to analyze the parameters of defects A and C, the directivity pattern should be altered. It can be achieved by selecting the one or more of the following options.

- The location of the transducer can be changed;
- The excitation frequency of a transducer can be changed;
- The type of wave mode (e.g., the S0, A0 or SH0 in low-frequency (LF) ultrasonic) can be changed;
- The utilization of the transducers array or different configuration of transducers can also be an alternative;

Therefore, its directivity pattern is one of the most important characteristics of a transducer. A prior information about the directivity pattern of a transducer before its usage can contribute to choosing a suitable excitation frequency at which a transducer must be excited and appropriate wave mode for the analysis of defects using ultrasonic non-destructive testing (NDT). It also leads to select the optimum number of transducers or configuration of transducers to be used for the testing of large and complex structures. This leads to reduce the overall cost of the SHM system. Moreover, the transmitting wave modes (the S0, A0 or SH0 in the case of LF ultrasonic) of interest can also be chosen as per requirements. Hence, the transmission/reception of unwanted wave modes can be avoided in order to minimise the coherent noise.

There are many theoretical, numerical and practical approaches developed for the analysis of directivity patterns of ultrasonic transducers [14,15,16,17,18]. Haig et al. estimated the directivity patterns of the S0, A0 and SH0 wave modes for macro-fiber composite (MFC) transducer glued on 10 mm thick steel plate using finite element (FE) modelling, and further validated by the experimental analysis [19]. In the latest research performed by Lowe et al. the behavior and directivity patterns of a flexible shear mode transducer were analysed by FE modelling and laboratory experiments for the SHM applications using LF guided waves [20]. Conjusteau et al. demonstrated the applicability of directivity information in the quantitative tomography and the construction of optoacoustic images [21]. Conjusteau et al. measured the spectral directivity of ultrasonic transducers with a laser point source in order to construct the optoacoustic image [21]. The required directivity can also be achieved by forming the array of transducers. Wang et al. found that the weaker directivity of micro-electromechanical systems (MEMS)-based capacitive ultrasonic transducer can be significantly increased by forming a linear array of such transducers [22].

The previously developed FE and experimental methods are limited to transducers of a specific shape and configuration, the dispersive characteristics of the propagation medium and the excitation frequency. Moreover, the numerical models and experimental analysis to predict the directivity patterns of a transducer takes much longer time as compared to the analytical approach. Therefore, a simplified, versatile and fast processing analytical model in order to predict the directivity pattern of any contact-type ultrasonic transducer in any medium of know dispersive characteristics was required to improve the ultrasonic NDT and SHM of the structures.

The objective of the presented research work was to develop an efficient and fast processing 2D analytical model for the estimation of directivity pattern of contact-type ultrasonic transducer by knowing the behavior of a transducer and dispersive characteristics of the propagating medium. The transducer may be of any shape and can be excited at any frequency. This work is the extension of a previously published research article in the conference proceeding [23]. The article is organized as follows. Section 2 demonstrates the procedure for the development of the 2D analytical model and its explanation by analyzing the P1-type macro-fiber composite (MFC) transducer glued on an Al alloy plate. The finite element (FE) modelling using *ANSYS* for the verification of results is explained in Section 3. The experimental approach with special scanning and signal processing methods to estimate the directivity characteristics (only for the A0 Lamb mode) is described in Section 4. The directivity patterns obtained by the developed analytical and numerical modelling along with its comparison with the experimental results are demonstrated in Section 5. Finally, the conclusions of the research are presented in Section 6.

## 2. Development of 2D Analytical Model

A versatile and simplified 2D analytical model based on Huygens’s principle was developed. The model can estimate and predict the directivity patterns of arbitrary contact-type GW transducers in any propagation medium, at any excitation frequency, and at any distance assuming that the behavioral characteristics of transducer and dispersive characteristics of the propagating medium are known. Based on the Huygens’s principle proposed by C. Huygens, the points on the wavefront can be assumed as the sources of wavelets which travel with the velocities of excited wave modes [24,25]. The basic methodology and specific underlying assumptions associated with the analytical modelling are described as follows:

- The ultrasonic contact type transducer of any shape and size can be assumed as uniformly distributed point sources on a 2D surface.
- Create the equally spaced arbitrarily receiving points (elements) along the half-circle (0° to 180°) at the required distance (radius) from the center of the transducer (origin).
- Calculate the distance vectors from all point sources to each arbitrary receiving element and integrate them.
- Compute the theoretical phase velocity characteristics of the propagating modes in a medium by the “Disperse” [26] computational package.
- Calculate the transfer function which is the multiplication of phase and attenuation components and depends on the excitation frequency, distance vector and the phase velocity of the propagating wave modes.
- The input signal is necessary to be multiplied by the correction factor (amplitude factor and direction factor). The amplitude factor depends on the type of propagating modes (e.g., the S0, A0, and SH0 in LF ultrasonic) and describes the signal distribution along the structure of transducer. The direction factor corresponds to the direction of propagating wave modes. The detailed description of the correction factor is discussed in Section 2.1.
- Take an account the diffraction in distances and calculate the distance diffraction factor.
- Calculate the output spectrum by multiplying the transfer function, corrected input signal spectrum (Fourier transform of the input signal after multiplying by correction factor) and the distance-dependent diffraction factor.
- Calculate the output signal (B-scan or series of A-scans) in time-domain to estimate the directivity pattern of a transducer (by plotting the normalized peak-to-peak amplitudes of the propagating particular modes in the polar coordinates).

### 2.1. Description of 2D Analytical Modelling

**Figure 2:**
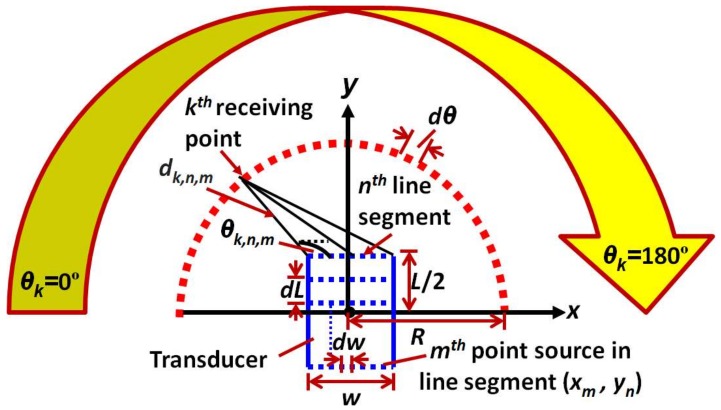
Description of 2D analytical modelling.

The description of the model is illustrated in Figure 2. It should be noted that a transducer with a rectangular shape is analyzed in the analytical model. However, the basic principle entirely depends on the calculation of distances between the point sources on the 2D surface of the transducer and the arbitrary receiving points. Thus, the model will work for the transducer possessing any shape and configuration. The two-dimensional (2D) coordinate system was used in modelling and origin was assumed to be at the center of the transducer. The length (*L*) and width (*w*) of the rectangular transducer section are in the direction along the *y*-axis and *x*-axis respectively.

The transducer section was divided into *n* different line segments (*n* = 1, 2 ... *N*) with the step size of *dL* along its length (*L*). In order to form the point sources along the 2D structure of a transducer, each of the line segments was split into a series of *m* points (*m* = 1, 2 ... *M*) with the separation distance of *dw* along the width. The coordinates of the transmitting point sources (*x_m_*, *y_n_*) are described as [23]:

(1) $$x_{m}=(m-1)\cdot dw-w/2,y_{n}=(n-1)\cdot dL-L/2$$

It should be noted that high resolution in the results (directivity patterns) can be achieved by keeping the *dL* and *dw* as shorter as possible. On another hand, simulation time of the model can be reduced with higher values of the *dL* and *dw*. Therefore, an optimal value can be selected depending on the spatial size of transducer considered for the modelling.

The *k* arbitrary receiving elements (*k* = 1, 2 ..., *K*) were created along the half-circle of radius R from the origin (center of the transducer) to construct the directivity pattern in the polar coordinates. If *θ_k_* is the angular position of receiving points along the half circle (0° to 180°) with respect to the origin (*θ_k__=_* [(*k*−1) ∙*dθ*], *dθ* is the angular step), the coordinates of receiving elements can be expressed as [23]:

(2) $$x_{k}=R\cdot \cos(\theta_{k}),y_{k}=R\cdot \sin(\theta_{k})$$

In the next step, the distance between the point sources to each receiving point was calculated. The distance vector (*d_k,n,m_*) to the *k*th receiving element from the *m*th point source presented on the *n*th line segment of the transducer and the respective angles (*θ_k,n,m_*) were calculated as follows:

(3) $$d_{k,n,m}=\sqrt{{\left(x_{k}-x_{m}\right)}^{2}+{\left(y_{k}-y_{n}\right)}^{2}}$$

(4) $$\theta_{k,n,m}=\tan^{-1}\left[\frac{y_{k}-y_{n}}{x_{k}-x_{m}}\right]$$

The transfer function *H* (*f*, *d_k,n,m_*, *V_ph_*) was estimated as [27]:

(5) $$H_{T}(f,d_{k,n,m},V_{ph})=e^{-\alpha (f)\cdot d_{k,n,m}}\cdot e^{-j\left(\frac{2\cdot \pi \cdot f\cdot d_{k,n,m}}{V_{ph}(f,h)}\right)}$$

where *α*(*f*) is the attenuation coefficient which is frequency (*f*) dependent and can be assumed to be zero for the isotropic lossless medium, *V_Ph_* is the phase velocity which depends on the excitation frequency (*f*) and the thickness (*h*) of the propagation medium.

Depending on the propagating GW wave modes, the correction factor (*C_F_*) to be multiplied to the input excitation signal *u_E_*(*t*) can be calculated as follows:

(6) $$C_{F}=A_{F}\cdot D_{F}(\theta_{k,n,m})$$

where *A_F_* is the amplitude factor which depends on the signal distribution along the structure of a transducer, *D_F_* is the direction factor which is the function of *θ_k,n,m_* for a specific GW mode.

The corrected input signal (*u_EC_*(*t*)) corresponding to the particular GW mode can be calculated as:

(7) $$u_{EC}(t)=u_{E}(t)\cdot C_{F}$$

The output signal in the frequency domain at *k*th receiving element is then calculated as:

(8) $$U_{R,k}(f,\theta_{k})=\sum_{n=1}^{N}\sum_{m=1}^{M}U_{EC}(f)\cdot H_{T}(f,d_{k,n,m},V_{p}{}_{h})\cdot \frac{1}{\sqrt{d_{k,n,m}}}$$

where *U_EC_(f)* is the spectrum of the corrected input signal *u_EC_(t)* which is calculated by obtaining the Fourier transform (*U_EC_(f)* = *FT [u_EC_(t)]*)*, U_R,k_* (*f, θ_k_*) is the spectrum of the received signal at *k*th receiving element, *θ_k_* is the angle in degree at *k*th receiving point and 1/√*d_k,n,m_* is the distance diffraction factor [28].

The inverse Fourier transform (FT^−1^) is then applied to construct the waveform of the received signal at *k*th receiving element in the time domain:

(9) $$u_{R,k}(t,\theta_{k})=FT^{-1}[U_{R,k}(f,\theta_{k})]$$

The peak-to-peak amplitudes (*A_pp_*(*θ_k_*)) and the normalized peak-to-peak amplitudes of the *A-scan* signals (*A_npp_*(*θ_k_*)) at the receiving points can be calculated as follows:

(10) $$A_{pp}(\theta_{k})=\max[u_{R,k}(t,\theta_{k})]-\min[u_{R,k}(t,\theta_{k})]$$

(11) $$A_{npp}(\theta_{k})=\left[\frac{A_{pp}(\theta_{k})}{\max[A_{pp}(\theta_{k})]}\right]$$

The normalized amplitude *A_npp_(θ_k_)* can be plotted in the polar coordinate system by varying the angle *θ_k_*, from 0° to 180° in order to construct the directivity pattern for a specific GW mode.

### 2.2. Demonstration of Analytical Model in the Case of P1-Type MFC Transducer

In order to demonstrate the model for practical applications, the MFC transducer manufactured by Smart Material Corporation [29] was analyzed to estimate the directivity patterns by the proposed analytical model. The MFC consists of rectangular piezoceramic fiber rods, which are sandwiched between layers of electrodes. These electrodes basically make an interdigitated pattern so that voltage can be transferred to and from the rods. As MFC is a thin, low weight, flexible and easily molded small sheet, it can be mounted/glued to the surface of the object or embedded into the multi-layered composite structures of arbitrary configuration. Generally, MFCs operate in two modes: d33 mode or elongation and d31 mode or contraction [29]. In this research work, P1-type MFC transducer (M-2814-P1) with dimensions (28 × 14 mm) was selected which operates in d33 (elongation) mode and can actuate and sense ultrasonic waves along the length of the MFC. One of the best features of MFC transducer is that MFC is an efficient transmitter and receiver of the asymmetric (A0) and the symmetric (S0) modes of guided Lamb waves at LF ultrasonic [30,31]. Apart from the fundamental Lamb wave modes (the A0 and S0), the fundamental shear horizontal mode (SH0) is another kind of guided wave which is completely non-dispersive [19]. The S0 (in the direction of wave propagation) and SH0 (in the perpendicular direction of wave propagation) modes are mainly distributed *in-plane* and the A0 mode is *out-of-plane*. As P1-type MFC operates in elongation mode, the S0 mode is the most dominant.

**Figure 3:**
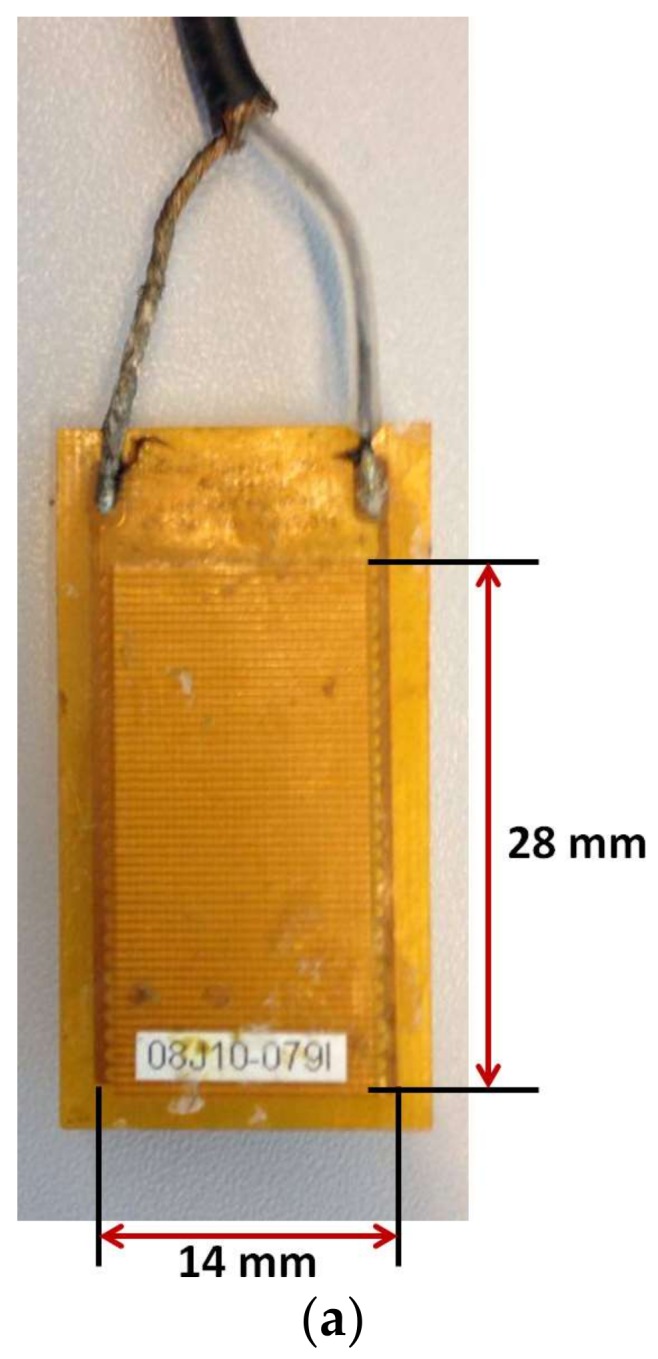

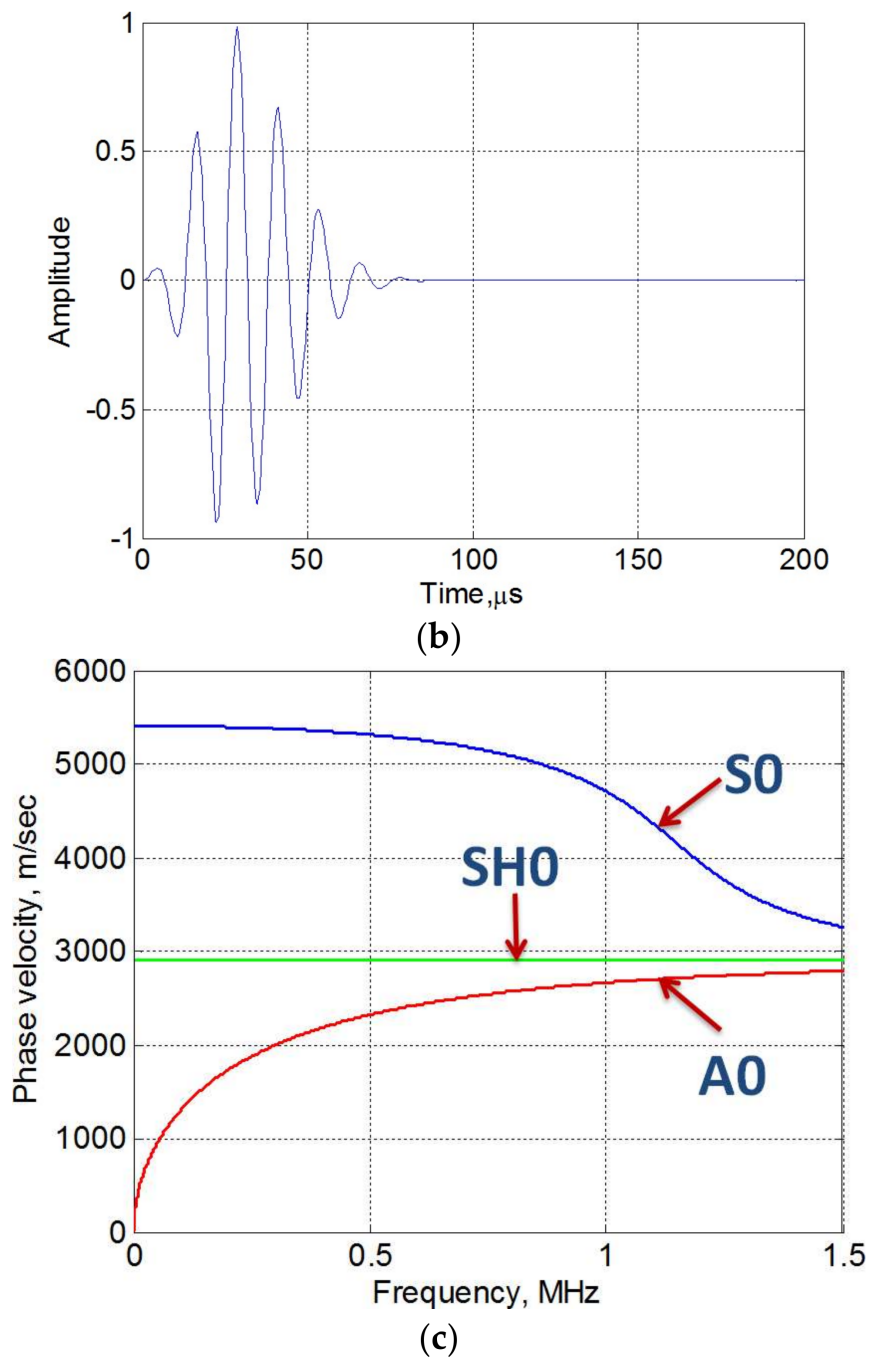
MFC transducer and guided waves: photo of P1-type MFC transducer (**a**), excitation signal of 80 kHz, 5-period burst (**b**) and phase velocity dispersion curves of GW modes in 2 mm thick Al alloy plate (**c**).

The task was to calculate the directivity of P1-type MFC transducer glued on 2 mm thick aluminum (Al) alloy plate at the distance of 300 mm from the center of MFC transducer in the generation of GW modes (the S0, A0, and SH0). The plate of Al alloy was assumed to be infinite dimensions, therefore the length and width of it are not considered. The transducer was excited using 80 kHz, 5-period burst signal with a Gaussian envelope and the sampling frequency of 1.6 MHz was used to record the received samples. A photo image of the MFC transducer, excitation signal and the phase velocity dispersion curves of the GW modes in 2 mm Al plate are shown in Figure 3. The phase velocities of the S0, SH0 and A0 modes at 80 kHz excitation frequency were estimated as 5409 m/s, 2903 m/s, and 1183 m/s respectively.

**Figure 4:**
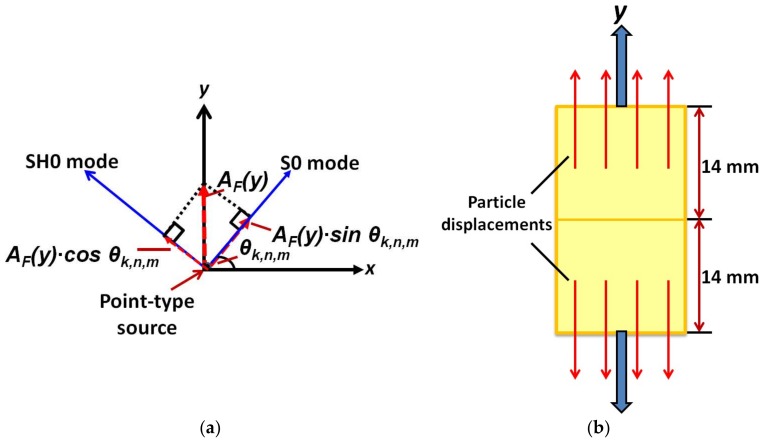
Calculation of correction factor (*C_F_*) for P1-MFC transducer: The particle displacements in excited P1-type MFC transducer (**a**), The correction factor *C_F_* (multiplication of the amplitude factor (*A_F_*) and direction factor for a specific GW mode (A0 or S0)) (**b**).

The MFC transducer was divided into 29 equal segments along its length (28 mm) with a separation distance of 1 mm. Each segment was analyzed as the 15 point sources along the width (14 mm) of MFC which lead the total number of point sources along the structure equal to 435. On another hand, total 181 arbitrary receiving elements placed at a distance of 300 mm from the center of MFC were located along the half-circle at the angular positions varying from 0° to 180°. The attenuation coefficient *α*(*f*) was assumed to be *zero* for the Al (isotropic and lossless medium) plate. The correction factor (*C_F_*) to be included for the specific mode is described in Figure 4. Figure 4a illustrates that the particle displacements in both halves of the P1-type MFC are in opposite direction along its length (in *y*-axis) due to its operation in elongation (d33) mode. Hence the approximated value of the amplitude factor (*A_F_*) was assumed to be equal to 1 and −1 at the edges of MFC (all point sources of first and last line segments) along its length (*y*-axis) which was decreased linearly towards the axis (center of MFC) where its value was set to *zero*.

For the estimation of the direction factor (*D_F_*), the theory of GW modes and the behavior of transducer were utilized. If *θ_k,n,m_* is the angle between the point source and a receiving element, the direction factor (*D_F_*) for the S0 and SH0 will be calculated by *sin* (*θ_k,n,m_*) and *cos* (*θ_k,n,m_*) respectively and illustrated in Figure 4b. The direction factor *D_F_* for the *out-of-plane* A0 mode will be *unity*. Therefore, the correction factor (*C_F_*) for each mode was calculated from Equation (6) as [32,33]:

(12) $$C_{F}(S0)=A_{F}(y)\cdot \sin(\theta_{k,n,m}),C_{F}(SH0)=A_{F}(y)\cdot \cos(\theta_{k,n,m}),C_{F}(A0)=A_{F}(y)$$

## 3. Numerical Simulation Using FE

The numerical simulation was performed by developing the finite element (FE) model using the “ANSYS” commercial software for the verification of analytical results. The 3D model of Al-alloy plate (1000 × 1000 × 2 mm) with the mechanical properties incorporated in Table 1 was developed using *SOLID 64* elements which are widely used for three-dimensional modelling of solid structures. Each element contained 8 nodes with a degree of freedom (DOF) equals to three: translation in *x*, *y* and *z* directions. Each element size was 1 mm. It should be noted that MFC was not modeled explicitly and only the behavior of the MFC was analyzed in order to develop the transducer section on Al alloy plate [34]. The appropriate force (excitation signal) of a five-period burst and frequency of 80 kHz with the Gaussian envelope (Figure 3b) was applied for investigating the wave propagation effects at the distance of 300 mm from the center of MFC transducer.

**Figure 5:**
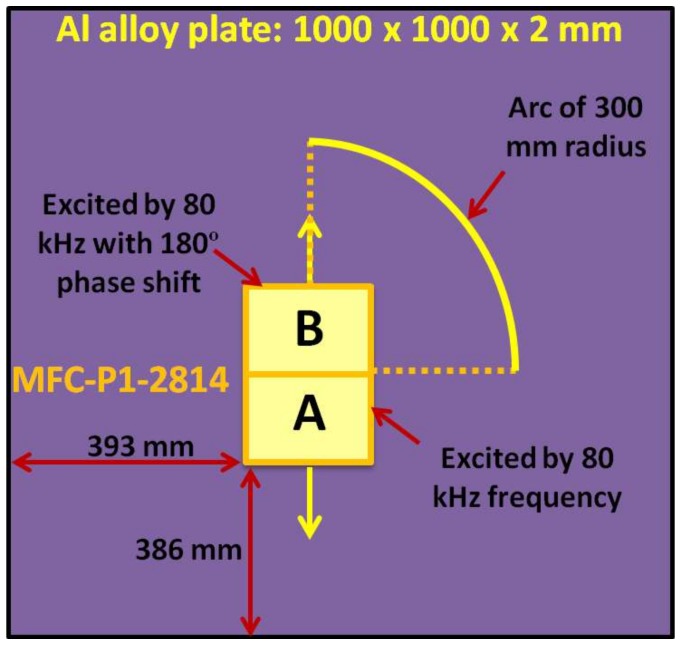
Description of numerical modelling of P1-type MFC transducer glued on Al alloy plate using FE.

For the calculation of directivity pattern, it was required that excitation zone and its behavior should be similar to the MFC-P1-2814. As the MFC-P1-2814 works in elongation or d33 mode, the upper and lower half of MFC transducer (28 × 14 mm) was excited by 80 kHz signals but in opposite phase. The sampling frequency was kept at 1.6 MHz. Due to symmetry associated with MFC structure and for shortening the simulation time, only a quarter-circle of 300 mm radius was selected to locate the receiving elements as illustrated in Figure 5. The transmitting zone (MFC) was positioned at 393 mm distance from the left side and at 386 mm distance from the bottom side of Al plate. The post-processing and the node selection to develop the quarter arc of 300 mm radius were performed in *MATLAB*.

**Figure 6:**
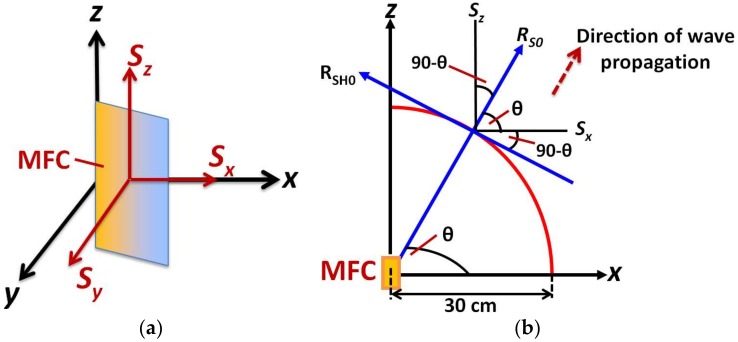
Calculation of resultant signals in FE numerical modelling: Showing signal components of particle velocity (*S_x,_, S_y_* and *S_z_*) along the axis (**a**) and resultant/received signals in the direction of propagation (*R_S0_*) and perpendicular to the direction of propagation (*R_SH0_)* (**b**).

The solution was obtained using numerical integration and in this way, the particle velocity distribution in the whole simulated structure at different time instants was obtained. To construct the directivity pattern of S0 mode and SH0 mode, the resultant particle velocity signals (*in-plane*) in direction of propagation and in the perpendicular direction to the propagation had to be determined. The *out-of-plane* signal to construct the directivity pattern of the A0 mode was the same as a y-component signal of the ANSYS model. The calculation of resultant signals to construct the directivity pattern is presented in Figure 6.

Let assume, that *S_z_*, *S_x_*_,_ and *S_y_* are the three components of received particle velocity signals in the z-direction (along with the length of MFC), x-direction (along with the width of MFC) and in the y-direction (*out-of-plane*) respectively at the receiving points along the arc of 30 cm in *ANSYS* 3D modelling. These signal components are shown in Figure 6a. It must be noted that resultant *out-of-plane* signal (*R_A0_*) to construct the directivity pattern of A0 mode was equal to the *S_y_*_._ The *in-plane* resultant signals in the direction of propagation (*R_S0_*) and perpendicular to the direction of propagation (*R_SH0_*) were calculated by analyzing the projection of *S_z_* and *S_x_* on *R_S0_* and *R_SH0_* respectively. The graphical representation to calculate these signals are shown in Figure 6b and can be described as follows [25,26]:

(13) $$R_{S0}=S_{z}\cdot \sin\theta +S_{x}\cdot \cos\theta$$

(14) $$R_{SH0}=S_{z}\cdot \cos\theta -S_{x}\cdot \sin\theta$$

(15) $$R_{A0}=S_{y}$$

where *θ* is the angle in degree from positive *x*-axis to the direction of wave propagation.

**Figure 7:**
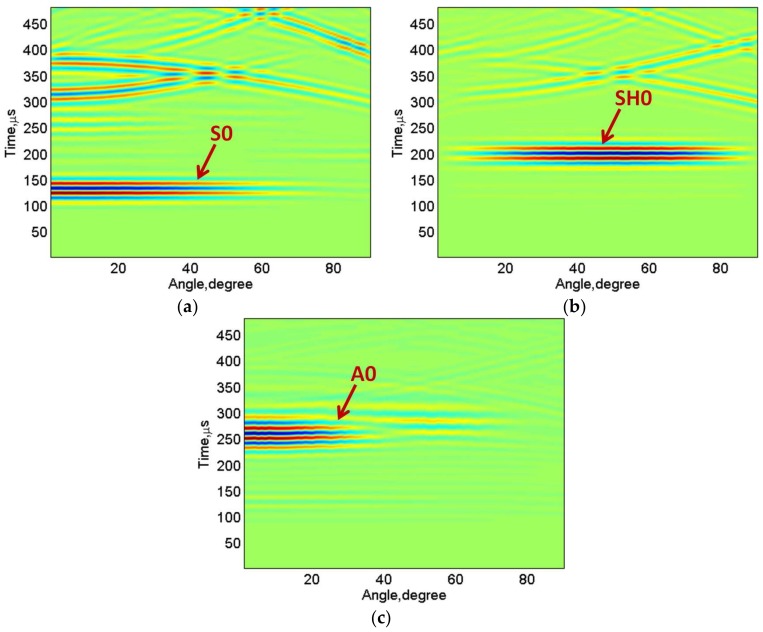
The B-scan images of particle velocity components in the direction of propagation (the S0) (**a**), perpendicular to the direction of propagation (the SH0) (**b**), and in the direction of propagation (the A0) (**c**).

The B-scans images of the S0 (the signal *R_S0_*), SH0 (the signal *R_SH0_*) and A0 (the signal *R_A0_*) waves were acquired in the computational package MATLAB and shown in Figure 7a–c. It can be clearly observed that the S0 mode was the fastest with an approximate time of arrival of 100 μs and the A0 was the slowest with an approximate time of arrival 225 μs. By applying the appropriate time windows on the respective B-scans, each mode (the S0, A0 or SH0) can be extracted to construct the directivity patterns.

## 4. Experimental Investigation

In order to validate the results obtained by the analytical and FE modelling, the experimental investigation was performed to estimate the directivity of P1-type MFC transducer glued at the center of Al alloy plate. The mechanical properties and the geometry of the Al alloy plate were similar as described in the part of FE modelling (Section 3). The signal of 5-period burst, amplitude of 20 V and frequency of 80 kHz was used to excite the MFC transducer. During the experiments, the point-type wideband ultrasonic transducer with a conical protection layer (diameter < 0.3 mm) and bandwidth up to 350 kHz at −6 dB level was used as a contact type receiver to receive the GW modes at 100 MHz sampling frequency with minimum distortions [11]. The mechanical scanning unit was used for the positioning of the receiving transducer in the polar coordinate system.

**Figure 8:**
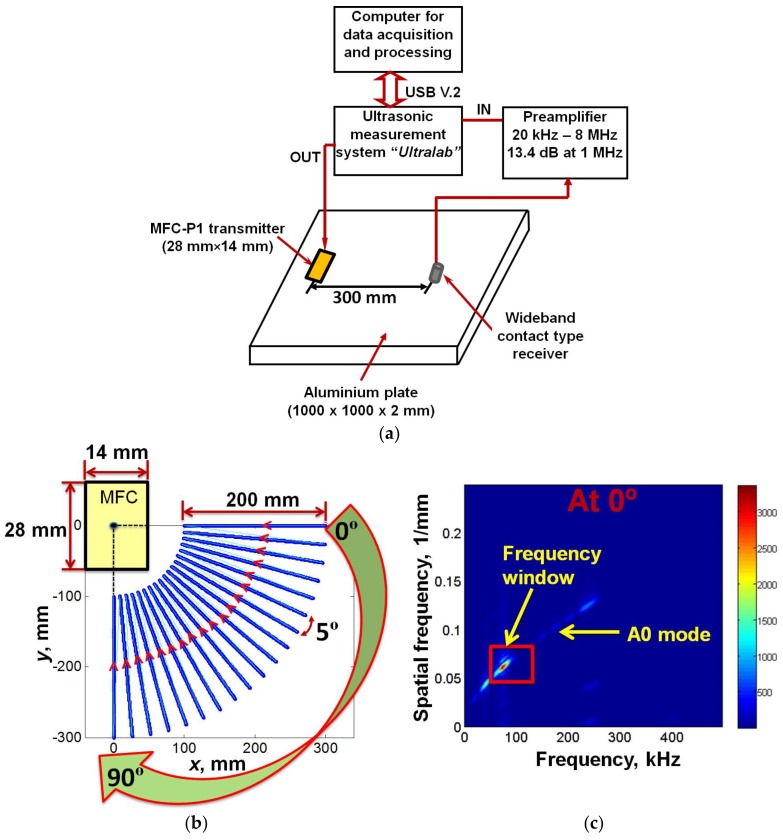

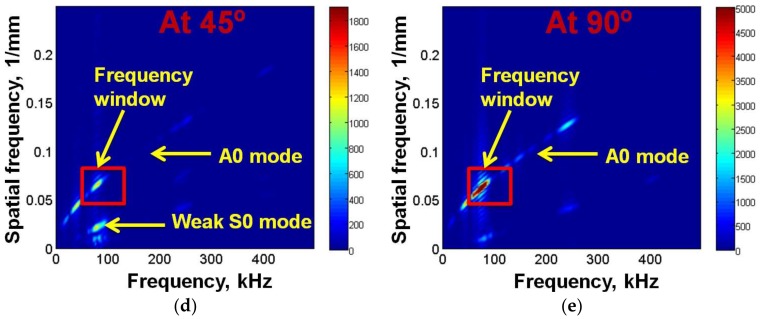
Experimental analysis and post-processing: Experimental set-up showing the glued MFC on Al alloy plate and the initial position of a point-type contact transducer (dimensions are not in scale) connected with LF ultrasonic system (**a**), showing the scanning process to acquire 19 B-scans at each 5° in the angular direction from 0° to 90° (**b**), showing the application of frequency window on the dispersion curves of the first (at 0°), tenth (at 45°) and nineteenth (at 90°) B-scans for selecting the appropriate signals at excitation frequency of 80 kHz (**c**–**e**).

As the point-type receiver operates in thickness mode, the A0 mode in comparison to other modes was captured more effectively. Therefore, during experiments, the directivity of only A0 mode was estimated for the comparative analysis with the modelling results. The experimental set-up is shown in Figure 8a. The experiment was performed using the Ultralab low-frequency (LF) ultrasonic measurement system, which has been developed at Ultrasound Institute of Kaunas University of Technology.

The pitch-catch technique (MFC transmitter was fixed and receiving transducer was scanned) was performed for the experiment as only one side of the object had to be accessed during the experimental investigation. The receiving transducer was initially positioned at 300 mm from the MFC along its width. The received signals were acquired along the quarter circle (0 to 90°) to construct the directivity pattern of the A0 mode. In order to capture the GW modes with minimum interference of neighboring modes and minimum distortion, a special scanning procedure and signal processing was used as illustrated in Figure 8b–e. The entire process can be described as follows:

- The quarter circle having the radius of 300 mm and the angular direction from 0 to 90° was divided into the 19 points located at each 5° as shown in Figure 8b. The linear scanning was performed by moving the contact-type transducer up to the 200 mm with a scanning step of 0.5 mm from each point towards the MFC transducer.
- In this way, 19 B-scan images were recorded in total within the angular direction from 0° to 90°. The sampling frequency of 100 MHz was used.
- The two-dimensional Fourier transform (2D-FFT) was applied to each B-scan for the transformation of measurements in distance-time into the spatial frequency-frequency [32,35]. Figure 8c–e shows the dispersion curve generated after applying the 2D-FFT on first B-scan (at 0°), tenth B-scan (at 45°) and nineteenth B-scan (at 90°).
- Although the propagation medium (Al alloy) is isotropic, the MFC is not a point-type transducer and generates not only the fundamental guided Lamb modes (the S0 and A0) but the non-dispersive GW mode (the SH0) is also generated. Therefore, the dispersive characteristics obtained for all 19 B-scans were not similar. In order to select the signals of interest, a rectangular window within the frequency range of 60–110 kHz and spatial frequency range of 0.04–0.075 1/mm was applied on each of the transformed B-scan signal (2D-FFT). The window was selected in such a manner that possible values of signal energies in each case were contained. The application of frequency window on the dispersion curves of the first (at 0°), tenth (at 45°) and nineteenth (at 90°) B-scans are shown in Figure 8b–e.
- Finally, the maximum energy of all 19 signals in the selected window was estimated in order to construct the directivity pattern in the polar coordinates (from 0° to 90°).

The experiment and the signal processing was repeated for the excitation signal of 220 kHz, 5-period.

## 5. Results

**Figure 9:**
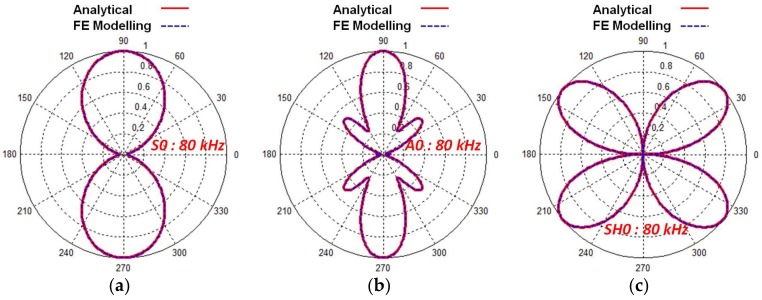
Normalized directivity characteristics of the S0 (**a**), A0 (**b**) and SH0 (**c**) guided wave modes at 80 kHz, 5-period excitation signal for the P1-type MFC transducer glued on 2 mm Al alloy plate obtained by analytical and FE modelling.

The directivity characteristics of GW modes possessing *in-plane* components (the S0 and SH0) and *out-of-plane* components (the A0) were estimated at 80 kHz, 5-period excitation signal in the case of P1-type MFC transducer glued on 2 mm Al alloy plate. The normalized directivity characteristics obtained from the analytical modelling (Section 2.2) and FE (Section 3) analysis were exactly matched as shown in Figure 9. It should be noted that directivity patterns were constructed and plotted in the polar coordinates from 0° to 360°. The symmetry of the MFC structure facilitated to utilize the mirror image of pattern across the axes. It means that directivity characteristics calculated in the polar coordinates from 0 to 90° are similar to those calculated from 270° to 360°. In a similar way, the directivity characteristics obtained in the polar coordinates from 0° to 180° would be similar to those calculated in the range from 180° to 360°.

The P1-type MFC operates in the elongation or d33 mode. Hence, the GW mode in the direction of propagation (the S0) is most dominant and directional in comparison to the A0 and the SH0 mode (Figure 9a–c). As shown in Figure 9a, the most of the wave energy associated with the S0 and A0 modes was observed towards 0° and 90° (longitudinal or in the direction of propagation). However, the A0 mode contained the side lobes with significant beam-width at 40°, 140°, 220° and 320° and the smaller side lobes at 0 and 180° apart from the major lobes (Figure 9b). The beam-width of major lobes in the case of A0 was also narrower as compared to the S0 mode. The number of wavelengths (*λ*) or signal distributions along the dimension of the transducer has an important role in the shape of directivity patterns. For the 80 kHz frequency, the wavelength for the S0 mode (*λ* = 67 mm) is almost 2.5 times of the length (28 mm) of MFC transducer. On another hand, it is equal to almost half of the length of MFC (*λ* = 14.83 mm) in the case of A0 mode. Although SH0 travels along the perpendicular direction of wave propagation (along the width of MFC) but upper and lower halves of MFC vibrating in opposite directions suppress the wave intensity along the 0° and 180° and therefore the SH0 waves possess the wave intensities at 40°, 140°, 220° and 320° in the resulting pattern (Figure 9c). Thus, the specific directivity characteristics of the SH0 mode are necessary to be taken into account during application of P1-type MFC transducers in NDT tasks.

**Figure 10:**
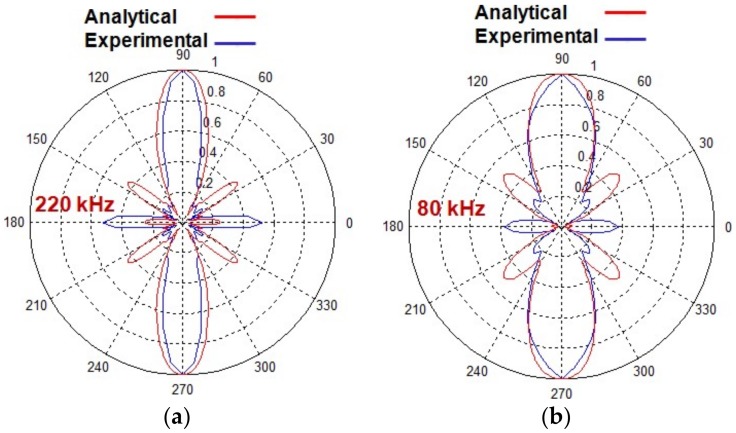
Comparative analysis of normalized directivity patterns of the A0 wave mode obtained by analytical modelling and experimental investigation at 80 kHz, 5-period excitation signal (**a**) and 220 kHz, 5-period excitation signal (**b**).

The experimental result of the directivity pattern (only for the A0) was obtained at 80 kHz and 5-period excitation signal in order to perform the comparison with the results obtained by analytical modelling. The comparative result is shown in Figure 10a. The analytical results exhibit the sufficient resemblance with the results obtained from the experimental investigation. The same number of lobes (2 major lobes at 90° and 180° and 6 side lobes at 0°, 40°, 140°, 220° and 320°) is visible in both patterns. The size and shape of the major lobes in both analytical and experimental results were almost similar but the significant variation in the size of side lobes was observed. At 0° and 180°, side lobes possessing more wave energy was observed in the experimental directivity pattern as compared to the analytical result. On another hand, the analytical results exhibited the more wave energy of side lobes at 40°, 140°, 220° and 320°.

The experimental analysis described in Section 4 was repeated again by exciting the MFC transducer at 220 kHz and 5-period excitation signal. The comparison of the experimentally and analytically obtained directivity patterns for the A0 mode at 220 kHz is shown in Figure 10b. The shape of major lobes and number of lobes of both directivity patterns are sufficiently matched but as observed in Figure 10a, the size of side lobes does not show the significant similarities. However, the analytical result again shows the good compromise with the experimental result. Although beam width of the major lobe at 220 kHz (Figure 10b) is narrower as compared to that of 80 kHz (Figure 10a), the number of side lobes is increased. The number of side lobes in the case of 220 kHz was observed as 10. The difference between the analytical and experimental results is due to the fact that the signal distributions (Amplitude correction factor (*A_F_*)) along the structure of MFC transducer were approximately considered depending on the behaviour of the transducer. The consideration of wave patterns (signal wavelengths) along the structure of transducer could improve the results of analytical modelling in future research.

## 6. Conclusions

The fast processing 2D analytical model has been developed in order to predict the directivity patterns of contact type ultrasonic transducers by knowing the behavior of the transducer and the dispersive characteristics of the propagating medium. The model is valid to estimate the directivity pattern at any distance excitation frequency. The proposed model will not only facilitate to select the position and the number of transducers but also leads to selecting the specific transducer and wave modes for the inspection of a particular type of defects using ultrasonic NDT. The novelty of the presented research work can be summarized as follows:

- Development of a 2D analytical model of contact type transducers based on Huygens’s principle capable to draw the directivity patterns within a far shorter processing time as compared to the finite element modelling and experimental investigation.
- Explanation of model for p1-type macro fiber composite (P1-type MFC) transducer manufactured by Smart Material excited by 80 kHz, 5-period excitation signal. The propagation medium was 2 mm aluminum alloy plate. Verification of the results was performed using FE modelling.
- Special experimental scanning and signal processing approach was also proposed in order to estimate the directivity patterns of P1-type MFC transducer glued on 2 mm Al alloy plate and exciting the fundamental modes with the lower interference of additional reverberations.

The directivity patterns of the S0, A0 and SH0 modes generated by P1-type MFC transducer at 80 kHz frequency were perfectly matched with the results obtained using FE modelling.

The directivity patterns of the A0 mode estimated by experimental investigation at 80 kHz and 220 kHz excitation frequencies were also similar to the analytical results except for the small differences in the beam-width of the main lobe and length of the side lobes. The reasons for differences are related to the signal distribution along the MFC structure and the amplitude correction factor (*A_F_*) assumed in the development of analytical modelling. The approximated magnitudes of amplitude correction factor were assumed in the analytical modelling in accordance with the expected behavior of the transducer. In the future research, the signal distributions along the structure of MFC transducer can be improved by analyzing the number of signal wavelengths of propagating fundamental modes in more details and therefore the accuracy for setting the values of amplitude correction factor can be increased. Moreover, it should be noted that the total simulation time of the proposed analytical model was significantly shorter in comparison to the time spent on experimental investigation and FE modelling.

## Figures and Tables

**Table 1:** Mechanical properties of Al-alloy plate.

Symbol	Quantity	Numerical Value
*E*	Young’s modulus	71 GPa
*ρ*	Density	2780 kg m^−3^
*υ*	Poisson’s ratio	0.33
